# When the war never ends: conceptual limitations, contextual differences and implications for interventions in continuous traumatic stress in Sub-Saharan Africa

**DOI:** 10.3389/fpsyt.2026.1909621

**Published:** 2026-09-22

**Authors:** Jane Namusoke, Betty Akurut Enyipu, Nathaniel Mayengo, Ronald Bahati, Kennedy Amone-P’Olak

**Affiliations:** 1 Department of Psychology, Faculty of Social Sciences, Kyambogo University, Kampala, Uganda; 2 Department of Postgraduate Studies, Counselling Psychology Program, Uganda Christian University, Mukono, Uganda; 3 Department of Foundations of Education and Educational Psychology, School of Education, Kyambogo University, Kampala, Uganda

**Keywords:** conceptual limitations, contextual differences, continuous traumatic stress, ecologies, interventions, violent conflicts, sub-Saharan Africa

## Abstract

Considerable knowledge has been generated on the symptoms, course, therapy, and care of survivors of traumatic experiences since posttraumatic stress disorder (PTSD) was added to the Diagnostic and Statistical Manual of Mental Disorders, third edition (DSM-III) in 1980. Notwithstanding this improved understanding, there is still disagreement over the definition, characteristics, and expression of PTSD in various settings. The aim of this paper was to offer a perspective on the limitations of the current conceptualization of mental health problems associated with continuous traumatic stress (CTS), considerations of contextual and cultural differences, and their implications for interventions. Particularly, this perspective aimed to address the generalization of Western therapies in non-Western societies, as well as the limitations of traditional rituals and ceremonies, and their limitations in the treatment of mental illness in the context of unending violent conflicts in Sub-Saharan Africa (SSA). In many traditional societies in SSA, trauma is experienced and symptoms are appraised based on the culture of survivors, demonstrating the importance of context in the conceptualization of CTS and the design of interventions. Psychological therapies in the majority of Western societies are based on symptom reduction, which are limited in the context of CTS and traditional cultures. The perspective also discusses different ecosystems in the treatment of trauma using Western and traditional society approaches, as well as the unique nature of the violent conflicts in SSA, and suggests a way forward. Finally, we discuss whether Western evidence-based therapies that are locally and culturally grounded and traditional rituals and ceremonies are mutually exclusive, particularly in the context of CTS and mass trauma that uniquely affect individuals, families, communities, and different subpopulations.

## Introduction

Posttraumatic stress disorder (PTSD) is the most prevalent mental disorder in the aftermath of violent conflicts. Since the inclusion of PTSD in the Diagnostic and Statistical Manual of Mental Disorders, third edition (DSM-III) in 1980, considerable knowledge has been generated about its symptoms, trajectory, treatment, and care (1). This increased knowledge notwithstanding, the definition, characteristics, and manifestations of PTSD in different contexts continue to be contentious (2). The differences in the PTSD definitions between the Diagnostic and Statistical Manual of Mental Disorders, Fifth Edition Text Revision (DSM-5-TR) and the International Classification of Diseases, 11th Revision (ICD-11) demonstrate this contention (3, 4).

The DSM-5-TR and ICD-11 conceptualize PTSD somewhat differently. The DSM-5-TR organizes PTSD symptoms into four clusters: intrusion, avoidance, negative alterations in cognition and mood, and alterations in arousal and reactivity (3). ICD-11 uses a narrower formulation centered on re-experiencing the traumatic event in the present, deliberate avoidance, and a persistent sense of current threat (4). Nevertheless, the ICD-11 construct was developed to specifically capture the sequelae of new concepts of sustained, repeated, and inescapable trauma such as complex posttraumatic stress disorder (CPTSD) (1). The CPTSD diagnosis that includes the additional three symptom groups (e.g., affective, relationship, and self-concept changes) is an improvement of the narrowly defined PTSD diagnosis. According to the WHO development goals for ICD-11, a CPTSD diagnosis is clinically practical and has demonstrated acceptable psychodiagnostic properties in several research studies, including good differentiation from other disorders such as personality disorder with borderline type (5).

Despite these developments, CPTSD and ICD-11 remain disorders arising after exposure to extremely threatening or horrific past events. Moreover, their application becomes more complex when the source of the threatening or horrific events remains active because responses such as heightened vigilance or even relationship changes may represent both traumatic distress and a realistic adaptation to the continuing threat.

While PTSD has been studied internationally, its dominant conceptual and diagnostic frameworks were initially developed largely within North American and European psychiatric traditions (6). Global diagnostic categories facilitate communication, research comparisons, and access to treatment, but they do not eliminate the need to consider cultural and contextual differences in the experience and expression of distress. Culture may influence how symptoms are interpreted and appraised and the language used to describe suffering, the people approached for assistance, and the interventions considered legitimate. Evidence-based psychological interventions have demonstrated benefits among conflict-affected populations in SSA (7, 8). However, treatments focused on symptom reduction alone may be insufficient where traumatic stress continues to be generated by insecurity, displacement, poverty, disrupted livelihoods, and damaged social relationships such as in the Democratic Republic of Congo (DR Congo), South Sudan, and northern Nigeria. The central issue, however, is not about the origin of interventions (e.g., the West or SSA), but whether the interventions adequately respond to the cultural, social, and material contexts and the conditions in which the distress occurs.

Estimates of the frequency of PTSD vary widely from 0% to 74%, especially in SSA (9). This variation is mainly driven by measurement heterogeneity, population selection, and timing (e.g., studies conducted during conflicts). The pooled prevalence of likely PTSD is estimated to be 22%, 30% in people who have experienced conflict, and 8% in those who have not experienced conflict, according to a prior systematic review and meta-analyses (9). Current theories of PTSD are predicated on the notion that the traumatic events happened in the past (10). However, in conflicts such as those in DR Congo or South Sudan, people experience severe psychological effects of constantly being in danger, as opposed to responding to a prior occurrence, as in PTSD. Systemic violence, ongoing domestic abuse, and active violent conflicts are common drivers of continuous traumatic stress (CTS) (11). In addition, contrary to typical PTSD, CTS is persistent, putting victims in a continuous state of vigilance and survival.

Particularly in the Great Lakes region (Burundi, the DR Congo, Ethiopia, Kenya, Rwanda, South Sudan, Tanzania, Uganda, etc.), millions of people live in a time of continuous peril and uncertainty due to violent conflicts, forced relocation, and epidemics such as Ebola virus disease, Marburg virus disease, and monkeypox (mpox) (12). Consequently, these traumatic stressors present distinct difficulties for diagnosis and interventions. While PTSD has been well described and characterized, CTS is still underdeveloped in psychological frameworks, literature, and intervention designs despite its importance in a variety of global situations, including communities dealing with systematic violence and in war-torn areas such as the DR Congo, Somalia, South Sudan, and the Sudan, among others.

The aim of this paper was to discuss and shape the current debate on the limitations of the current conceptualization of mental illness, considerations of contextual and cultural differences, and their implications for interventions. Particularly, we aimed to address the generalization of Western therapies in non-Western societies and in traditional rituals and ceremonies and their limitations in the treatment of mental illness in the context of CTS. Western-based mental health interventions focus on symptom reduction, whose effectiveness in the context of CTS may fall short of reducing distress. Contextual differences in CTS and possible differences in traditional and cultural expressions, as well as their implications for interventions (6), are described. In addition, although Western therapeutic interventions have shown symptom reduction (e.g., 7, 8), hardly any follow-up studies have demonstrated long-term sustainability in the reduction of symptoms. Lastly, our paper also aimed to shed light on and recognize that traditional rituals and ceremonies in trauma treatment complement culturally adapted and contextually appropriate Western therapies. It is crucial to understand and frame key mental health concepts within specific cultural contexts and design interventions that may help people deal with CTS. Such knowledge and framework should not only reduce symptoms but also systemically empower individuals and families and enhance community resilience in the face of CTS.

The War-Affected Youth Survey (WAYS) study and similar studies conducted in northern Uganda and elsewhere in SSA provided the background to this perspective (13). The WAYS study is a longitudinal cohort study of the risks, protective factors, and trajectory of mental health problems in war-affected youths in northern Uganda since 2010, which has several publications.

## Characteristics and distinctive features of traumatic events in the West and SSA

Several distinct features characterize traumatic events that predispose to traumatic reactions in the Western world and SSA. First, the majority of traumatic events in Europe and North America have a relatively distinct beginning and end; therefore, the concept of ‘post’-traumatic stress disorder may be used with more certainty. On the contrary, in low- and middle-income countries, especially in SSA, protracted wars such as those in South Sudan, the Sudan, the DR Congo, and the Sahel region, lack definite ends (14). Consequently, the diagnosis of PTSD is a challenge because traumatic events are continuous and the affected populations endure extremely stressful situations that continually endanger their lives. For instance, in northern Uganda, post-war environments are rife with a high density of stressors such as gang violence, youth unemployment, stigma against formerly abducted children, land conflicts, domestic violence, and general insecurity even after the guns have fallen silent (15, 16).

Second, violent conflicts in SSA have a crippling effect on society, affecting millions of individuals, if not entire communities. Often, the available infrastructure is overwhelmed, humanitarian assistance is overstretched, and the social and traditional resources and the safety net required for coping are severely weakened (17, 18). Importantly, the violent conflicts in SSA target and destroy ways of life, including crucial social, cultural, and material resources that are essential for people to bounce back. Moreover, in spite of a myriad of humanitarian assistance and psychological interventions, recovery is often without structural absorption. Therefore, both individually and collectively, the fabric that binds society together and the traditional practices and grass roots social relations are shattered, dissociated, and disordered, making communities helpless and hopeless.

Third, the nature of the violent conflicts in SSA is unique and unconventional. Violence is perpetrated in communities, sometimes by people known to each other, and survivors, perpetrators, and victims are often difficult to distinguish (19–22). For instance, in the WAYS study in northern Uganda, both formerly abducted youth (perpetrators—involved in killings and mutilations) and those who were not abducted (victims—suffered atrocities at the hands of perpetrators) equally suffered from adverse mental health problems (19). This has significant implications for interventions to mitigate mental health problems among war-affected populations in SSA.

Fourth, various subpopulations were affected differently by the war in northern Uganda at individual (personal), family, and community levels. At the individual level, the war affected various subpopulations differently (e.g., abducted *vs*. non-abducted; born in or out of captivity; perpetrator, victims, or victim/perpetrator; and sexually abused girls *vs*. sexually abused child mothers, among others). However, the majority of studies simply lumped all war-affected people together. For example, in a previous study with war-affected girls with a history of sexual violence and with children as a consequence, those who were sexually abused and with children had higher odds of reporting depressive or anxiety symptoms, somatic complaints, stigma, and general dysfunction than those who did not report sexual violence and do not have children (23). At the community level, discrimination and stigma are widespread, thus adversely affecting coping with the traumatic reactions and undermining reintegration and mental health help-seeking (23).

Lastly, trauma is recognized in many African cultures not only as a behavioral or emotional issue but also as a spiritual and community one. The relationships between parties to a conflict or between the living and the dead are highlighted by this more expansive understanding of trauma (7, 8). For instance, despite the loss of loved ones, the primary causes of post-war stress for survivors of the war in northern Uganda were the deterioration of community values, which is still mourned more than the aspect of the violent conflict (7, 8).

Although some previous studies (e.g., Bolton et al. (7)) have shown that Western therapies are effective in reducing symptoms, their primary focus has been on symptom reduction. The focus on symptom reduction limits the effectiveness of Western therapies in the context of CTS, which keeps on generating more symptoms or exacerbating the existing ones.

This paper describes examples from Liberia, Mozambique, Rwanda, Sierra Leone, South Sudan, the Sudan, and Uganda. Examples of traditional rituals and ceremonies from these settings are illustrative rather than representative. Different communities in SSA may have their own traditional therapies for the treatment of trauma according to their culture, trauma appraisal, and meaning making. However, the common thread in the majority of traditional rituals and ceremonies is that they are directed toward promoting holistic healing, strengthening resilience, and building individual, family, and community capacities that enable survivors to cope with persistent stressors while fostering relationships, enhancing recovery, and improving wellbeing.

Whereas symptoms such as nightmares and intrusion are understood as basic characteristics of PTSD in the West, among the people of northern Uganda, these are mediated through a spirit medium, *Cen*, a spirit of the dead that comes back to haunt the living for killing them or because they have not been given a decent burial (24). Consequently, traditional rituals such as ‘*Mato Oput*’ ceremonies to exorcise spirit possession, ‘*Stepping on the egg*’ to stop and decontaminate evil spirits, and giving someone a decent burial have been practiced for a long time among the Acholi community in northern Uganda (24, 25). The Acholi ‘*Mato Oput*’ ceremony promotes healing by restoring social cohesion, bolstering community support networks, encouraging reintegration, and making use of easily accessible local traditional resources that help long-term recovery in addition to addressing war trauma (25).

In Mozambique, the *Timhamba* rituals and *Magamba* ceremonies conducted by specialized healers are used to counter spirit possession and address severe trauma and conflicts rooted in Mozambique’s civil war (26). On the other hand, in Liberia, the *Zoes* (traditional healers) treat mental illnesses in the communities (27, 28). In Sierra Leone, traditional rituals and cleansing ceremonies have been shown to work independently or together with Western therapies (29).

In addition, in South Sudan, the Dinka community uses the *Cieng* ceremony to offer a holistic approach to trauma treatment, addressing both the social and physical aspects of psychological trauma. Social healing involves rituals to address the origins of the illness, while physical healing uses herbal remedies to relieve somatic symptoms. Furthermore, the Dinka healing system is interactive, including not only the perpetrators and survivors but also the entire community. Consequently, the community participates in the diagnosis, prognosis, and course of treatment (30). Beyond addressing war trauma, the *Cieng* ceremony facilitates healing by restoring social cohesion, strengthening community support networks, promoting reintegration into productive social and economic roles, and utilizing accessible local resources that foster sustainable recovery (30). In order to promote healing and alleviate trauma, adopting a traditional Dinka ‘*Cieng*’ ritual aided in both individual reintegration and communal reconciliation for war survivors in South Sudan (30).

The ongoing war in the Sudan (since April 2023) is a humanitarian disaster, displacing approximately 13 million people, crippling health systems, and exacting untold suffering and psychological trauma, which is a typical example of CTS (31). In a cross-sectional analysis, Khalil et al. (32) studied subpopulation differences among Sudanese refugees and internally displaced and non-displaced people. The odds of experiencing PTSD were higher among refugees than in both internally displaced and non-displaced persons, lending credence to the need for tailored interventions for different subpopulations (32). Still in Sudan, Elhusein et al. (18) highlighted the fragility of the current mental health infrastructure, the health system, and the current population-level mental health needs. They also identified priority needs for a coordinated and context-appropriate intervention (e.g., community-led mental health interventions) for the population in the Sudan (18).

In neighboring South Sudan is another humanitarian calamity displacing millions, causing severe food insecurity and untold mental anguish. Lee et al. (33) studied the physiology of chronic conflict in South Sudan. Their results showed high prevalence of symptoms associated with traumatic reactions, widespread emotional dysregulation, and unusual levels of parasympathetic nervous system activity, all indicative of a highly stressed population with significant health risks (31, 33).

In Rwanda, after the 1994 genocide in which between 800,000 and 1,000,000 people were killed, a community-based traditional justice system known as the *Gacaca Courts* was adopted upon the realization that both the victims and the perpetrators were going to live in the same community (34). Based on the Kinyarwanda idea of ‘justice on the grass’, the tribunals aim to promote reconciliation, rebuild the social fabric of society, and restore justice. In these courts, trauma-informed approaches are critical for advancing transitional justice and sustainable peace, which requires a safe environment where damaged community capacities can be rebuilt based on sociotherapy aimed at sustainable peace in post-conflict contexts (35).

Consequently, in this perspective, Western evidence-based approaches and local culturally grounded ones are not mutually exclusive. For example, structured treatment approaches designed precisely for populations living under ongoing threat and displacement, such as narrative exposure therapy (NET/KIDNET), have been shown to be effective (36), although their long-term sustainability remains largely unknown. Another example is in Mozambique, where Western therapies are used together with traditional rituals performed by ‘*Tanyanga*’ (spirit medium, diviner, or healer) to treat survivors with psychological trauma (37, 38). Similarly, it is important to add that some traditional rituals are not uniformly protective. Particularly, traditional rituals may lead to stigma, exclusion, and gendered harms and may ignore personal distress and the long-term outcomes on the wellbeing of survivors. Overall, adapted, evidence-based individual treatments and community traditional rituals and ceremonies can be complementary, particularly for mass trauma.

## Implications for interventions

Although there is evidence of established therapies for traumatic stress in SSA (7, 8), their efficacy is constrained by many concerns. First, individual wellbeing and recovery are the main focus of Western therapies such as trauma-focused cognitive behavioral therapy (TF-CBT); however, in SSA, each individual is a part of the entire community (17). It follows therefore that recovery from traumatic experiences is not only personal but is also a communal and a spiritual process, with the community playing a significant role in providing the social and cultural capital needed for recovery and subsequent wellbeing. In a prior study, collective/community strengths, religious faith, family communication, support networks, and peer support were identified as protective resources that can counteract acculturative and resettlement stressors that might keep Somali refugee children and their families in the USA in the path of PTSD (39).

Second, the Acholi people in northern Uganda use the term ‘cen’ (pronounced ‘chen’) to describe spirit possession and haunting. It is thought to be the evil spirit of someone who died brutally or unfairly, coming back to haunt or possess the living in order to exact retribution or to make amends (40). Similarly, it is widely believed among the Acholi ethnic group that the spirit of the one who has not been given a decent burial will return to haunt the living (41). Consequently, mediation of trauma symptoms by a spirit medium such as *Cen* in northern Uganda may offer opportunities for interventions through carrying out traditional rituals for reconciliation and appeasement. In addition, *Mato Oput* among the Acholi ethnic group in northern Uganda is a community-based restorative justice and reconciliation ritual. The ritual, which literally translates to ‘drinking the bitter root of “*Oput*” tree’, marks the formal conclusion of a blood feud or conflict between two groups (e.g., families, communities, etc.) and replaces cycles of retaliation with forgiveness and healing (42). These broader conceptualizations of trauma may have important implications for interventions such as overcoming the sense of guilt and restoring relationships between parties in a violent conflict (e.g., *Mato Oput*) or between the living and the dead (*decent burial*), thus reducing both further conflicts and mental anguish (41).

Finally, cross-cutting challenges such as functional impairments and the deterioration of the social fabric and the pillars that used to hold societies together are often neglected (17), particularly in Western therapies. In traditional cultures like those in SSA, however, rebuilding the social structure and the pillars of society may help lessen the negative impacts of mass and continuous trauma.

## Suggestions for the future

Various interventions have been proposed for populations who continue to endure traumatic events such as protracted violent conflicts. First, to offer continuing psychological assistance, non-specialist paraprofessionals [such as community mental health first aiders (MHFAs)] can receive training in low-intensity interventions such as brief, manualized, and culturally appropriate methods to persons in psychological distress in the community. Low-intensity interventions include psychoeducation, mental health literacy, training using manualized brief techniques such as Teaching Recovery Techniques (TRT) (e.g., to support survivors in the aftermath of violent conflicts), e.g., Problem Management Plus (PM+) (43, 44), and psychological self-help interventions (31). In addition, community MHFAs and paraprofessionals could be trained to hold supportive conversations with individuals in psychological distress due to CTS. A local psychological first aider, who is familiar with the local context and culture and knows individuals undergoing distress within their locality, may have greater familiarity with the local context and culture more than does a psychiatrist or a psychologist from urban centers or the city. Moreover, a psychological first aider can flag distress or act as a referral pathway to more specialized care. Although low-intensity interventions such as PM+, TRT, and MHFAs are not unique to SSA, they are easily culturally adaptable, inexpensive, and can be easily used by paraprofessionals who are well versed with the local culture and context. For example, in Kenya, PM+ has been adapted for use in the local context (45). Similarly, in Uganda, MHFAs have been culturally adapted for use in the local context (46).

Second, psychoeducation and mental health literacy are needed for war-affected populations to recognize the signs and symptoms of stress and to manage stress in order to reduce the physiological effects of trauma in their bodies and minds. Third, it has been demonstrated that engaging in activities including meditation, dancing, funeral rites, and other rituals may reduce the symptoms of traumatic stress and other reactions that people who are experiencing CTS may have (25–29). Such activities have the potential to bring closure to traumatic experiences and are meaningful and relevant to the culture and traditional practices of survivors of wars in many parts of SSA such as Uganda, Liberia, Mozambique, Sierra Leone, and South Sudan.

Fourth, survivors of traumatic events are known to experience abnormally high levels of stress (47). The hypothalamic–pituitary–adrenal (HPA) axis is crucial for triggering hormonal reactions to stressful stimuli, and persistent exposure to traumatic events is linked to a series of biological changes related to this axis (47). Although the core stress response may be physiologically similar in all cultures, the interpretations, meaning, and coping experiences may vary from one culture to another. Recognizing these differences is critical to the design of interventions. Consequently, a context-based stress management program is vital for those who have experienced or are still experiencing chronic stress. Those who have survived traumatic events can readily learn stress management skills such as breathing exercises, meditation, and remaining calm and composed. Rebuilding damaged relationships envisaged in the traditional rituals and ceremonies has a profound impact on the physical body, often associated with returning one to a state of safety from a condition of tension, most common among war-affected populations. In a similar vein, survivors of traumatic events frequently struggle with substance use disorders. All of these stress management programs can be implemented by paraprofessionals under supervision.

Fifth, understanding deficits in social functions associated with traumatic experiences is crucial to the design of interventions. For example, restoring the livelihood of those affected by war both at the individual and community levels is vital for alleviating psychological distress and somatic complaints (e.g., headache, stomach ache, fatigue, etc.). More importantly, reconstructing the social fabric and pillars that once held societies together, such as traditional cultural practices, and strengthening cultural leadership, among others, may be more effective interventions than traditional Western-oriented therapies (34, 35). Unlike in Western therapies, possibilities exist of promoting social connection as a protective factor through peer-led conversations and group activities to promote socioeconomic wellbeing.

Finally, further longitudinal research is required to establish the long-term sustainability of culturally adapted Western therapies not only in non-Western cultures but also in traditional rituals and ceremonies for treating trauma, reducing stigma, and improving the wellbeing of war-affected populations.

## Conclusion

While PTSD and CPTSD are invaluable concepts in understanding the experiences of individuals who endured traumatic events, they may be limited for societies experiencing unending protracted violent conflicts. Comprehending the functional deficits associated with experiencing traumatic events and understanding the daily challenges that affect survivors, both at the community and individual levels, numerous stakeholders (e.g., individuals, families, the community, and cultural leaders) may be required in the design of interventions. Of critical importance is the reconstruction of the social fabric and pillars that once held societies together and are responsible for the diagnosis, prognosis, and care of survivors. In light of the complexity in the conceptualization of CTS in many theaters of conflicts in SSA where protracted traumatic events are dominant, a more contextualized and nuanced understanding of traumatic reactions may provide better treatment outcomes.

Interventions to aid war-affected communities in regaining security, normalcy, and control while promoting community involvement and mobilizing internal resources to fulfill their needs are essential (48). Where the diagnosis, prognosis, and treatment of mental health problems include the entire community; thus, interventions must be guided by conceptual clarity, contextual considerations, and community involvement. In many ethnic groups in SSA, healing is a systemic process that occurs at the individual, family, and community levels. Enhancing resilience, empowering individuals, and developing family and community capacities to help survivors deal with ongoing stressors are key pillars to sustaining the wellbeing of war-affected populations.

## Data Availability

The original contributions presented in the study are included in the article/supplementary material. Further inquiries can be directed to the corresponding author.
